# Current perspectives on cell-assisted lipotransfer for breast cancer patients after radiotherapy

**DOI:** 10.1186/s12957-023-03010-z

**Published:** 2023-04-17

**Authors:** Qiuwan Wu, Shuai Chen, Wuyun Peng, Donghan Chen

**Affiliations:** 1 The First Affiliated Hospital of Xiamen University, School of Medicine, Xiamen University, 55 Zhenhai Road, Siming District, Xiamen, Fujian 361003 P. R. China; 2 The Third Clinical Medical College, Fujian Medical University, Xiamen, Fujian P. R. China

**Keywords:** Cell-assisted lipotransfer, Radiotherapy, Adipose-derived stem cell, Breast cancer

## Abstract

**Background:**

Cell-assisted lipotransfer (CAL), a technique of autologous adipose transplantation enriched with adipose-derived stem cells (ADSCs), has the potential to improve cosmetic outcomes at irradiated sites. However, many concerns have been raised about the possibility of ADSCs increasing oncological risk in cancer patients. With the increasing demand for CAL reconstruction, there is an urgent need to determine whether CAL treatment could compromise oncological safety after radiotherapy, as well as to evaluate its efficacy in guiding clinical decisions.

**Methods:**

A PRISMA-compliant systematic review of the safety and efficacy of CAL in breast cancer patients after radiotherapy was conducted. The PubMed, Ovid, Cochrane Library, and ClinicalTrials.gov databases were comprehensively searched from inception to 31 December 2021.

**Results:**

The search initially yielded 1185 unique studies. Ultimately, seven studies were eligible. Based on the limited outcome evidence, CAL did not increase recurrence risk in breast cancer patients but presented aesthetic improvement and higher volumetric persistence in a long-term follow-up. Although breast reconstruction with CAL also had oncological safety after radiotherapy, these patients needed more adipose tissue and had relatively lower fat graft retention than the non-irradiated patients (*P* < 0.05).

**Conclusions:**

CAL has oncological safety and does not increase recurrence risk in irradiated patients. Since CAL doubles the amount of adipose required without significantly improving volumetric persistence, clinical decisions for irradiated patients should be made more cautiously to account for the potential costs and aesthetic outcomes. There is limited evidence at present; thus, higher-quality, evidence-based studies are required to establish a consensus on breast reconstruction with CAL after radiotherapy.

## Introduction

Breast cancer is the most commonly diagnosed cancer and the leading cause of tumor-related death in women [1]. Multimodal treatment approaches have substantially improved patient outcomes. Among these approaches, radiotherapy is especially recommended to be performed in high-risk patients after mastectomy and patients who received breast-conserving surgery [2]. However, radiotherapy causes breast tissue damage and then leaves sequela, such as contour deformity, fibrosis, or chronic pain [3, 4]. Based on long-term experiences, the implantation of adipose tissue, known as lipofilling or lipotransfer, is considered a helpful remedy to correct sequela [5]. Lipofilling is also used to improve the cosmetic results of other reconstruction techniques, such as implant-based or autologous tissue-based reconstruction [6, 7]. Nevertheless, the main drawback of lipofilling is the high absorption rate, which always leads to poor graft retention and patient dissatisfaction with unpredictable aesthetic outcomes [8, 9].

Adipose-derived stem cells (ADSCs) were first characterized in 2001 [10] and found to have a high proliferative capacity and multilineage differentiation potential. Thus, transplantation of ADSCs is considered a promising strategy that could improve fat graft survival and the volume retention of adipose tissue. On this basis, Matsumoto et al. proposed the cell-assisted lipotransfer (CAL) method in 2006 [11]. The method used autologous adipose tissue containing ADSCs, enriched from a freshly isolated stromal vascular fraction (SVF). SVF is the aqueous fraction derived from lipoaspirate enzymatic digestion or mechanical separation. CAL was initially applied to the cosmetic breast and facial augmentation in 2008, and then, this technology was found to be potentially used for breast reconstruction in breast cancer patients [12]. CAL has also been reported to improve cosmetic outcomes at irradiated sites [13] and minimize complications resulting from radiotherapy [14].

Numerous clinical trials and studies have documented the effects and oncological safety of CAL in breast cancer patients [15–18]; however, many concerns have been raised about the possibility of ADSCs increasing recurrence risk in cancer patients [19–21]. It was reported that ADSCs might interact with breast cancer cells [20] and promote the radioresistance of breast cancer cells via a paracrine pathway [22, 23]. In breast cancer patients after radiotherapy, the safety and efficacy of CAL are still uncertain. With the increasing demand for CAL in breast plastic surgery worldwide [24, 25], there is an urgent need to determine whether this treatment could potentially compromise oncological safety in patients after radiotherapy. Therefore, the current study aimed to examine the literature and current clinical trials on CAL to assess the safety and efficacy of this technique in breast cancer patients after radiotherapy.

## Methods

### Search strategy

This systematic review was performed in accordance with the Preferred Reporting Items of Systematic Reviews and Meta-Analyses (PRISMA) statement [26]. A comprehensive, reproducible electronic search of the PubMed, Ovid, Cochrane Library, and ClinicalTrials.gov databases from inception to 31 December 2021 was conducted. The search strategy and search syntax are presented in Table 1. Searches were not restricted by language or study type. To ensure that the search strategy did not miss relevant studies, bibliographies of identified studies and other relevant articles, including recent review articles, were searched manually.

**Table 1:** Database search

Database	Search syntax
PubMed	("breast"[Title/Abstract] OR "mamm*"[Title/Abstract] OR "milk gland"[Title/Abstract]) AND ("radiotherapy"[Title/Abstract] OR “radiotreatment”[Title/Abstract] OR “radiation”[Title/Abstract] OR “irradiation”[Title/Abstract]) AND (“lipofilling”[Title/Abstract] OR “lipo-filling”[Title/Abstract] OR “lipomodelling”[Title/Abstract] OR “lipograft”[Title/Abstract] OR “lipotransfer”[Title/Abstract] OR “lipostructuring”[Title/Abstract] OR “lipotransplant”[Title/Abstract] OR “lipo-transplant”[Title/Abstract] OR “lipoinjection”[Title/Abstract] OR “lipo-injection”[Title/Abstract] OR “lipoaspirate”[Title/Abstract] OR “fat” [Title/Abstract] OR “adipose”[Title/Abstract] OR “adipocyte”[Title/Abstract] OR “stromal vascular fraction”[Title/Abstract] OR “adipose-derived stromal cell”[Title/Abstract] OR “adipose-derived stem cell”[Title/Abstract] OR “cell-assisted lipotransfer”[Title/Abstract])
Ovid	(breast or mamm* or milk gland).ab. AND (radiotherapy or radiotreatment or radiation or irradiation).ab. AND (lipofilling or lipo-filling or lipomodeling or lipograft or lipotransfer or lipostrcturing or lipotransplant or lipo-transplant or lipoinjection or lipo-injection or lipoaspirate or fat or adipose or adipocyte or stromal vascular fraction or cell-assisted lipotransfer or adipose-derived stromal cell or adipose-derived stem cell).ab
Cochrane Library	((breast):ti,ab,kw OR (mammary gland):ti,ab,kw OR (milk gland):ti,ab,kw) AND ((radiotherapy):ti,ab,kw OR (radiation):ti,ab,kw OR (irradiation):ti,ab,kw OR (radiotreatment):ti,ab,kw) AND ((adipose):ti,ab,kw OR ("stromal vascular fraction"):ti,ab,kw OR (lipotransfer):ti,ab,kw OR ("adipose-derived stromal cell"):ti,ab,kw OR ("adipose-derived stem cell"):ti,ab,kw OR (fat):ti,ab,kw OR (adipocyte):ti,ab,kw OR (lipofilling):ti,ab,kw OR (lipostructuring):ti,ab,kw OR (lipomodelling):ti,ab,kw OR (lipograft):ti,ab,kw OR ("cell-assisted lipotransfer"):ti,ab,kw)
ClinicalTrials.gov	(breast[Condition or disease]) AND (‘adipose’ OR ‘stromal vascular fraction’ OR ‘lipotransfer’ OR ‘adipose-derivedstromal cell’ OR ‘adipose-derivedstem cell’ OR ‘fat’ OR ‘adipocyte’ OR ‘lipofilling’ OR ‘lipostructuring’ OR ‘lipomodelling’ OR ‘lipograft’ OR ‘cell-assisted lipotransfer’[Other terms])

### Eligibility criteria and study selection

**Fig. 1:**
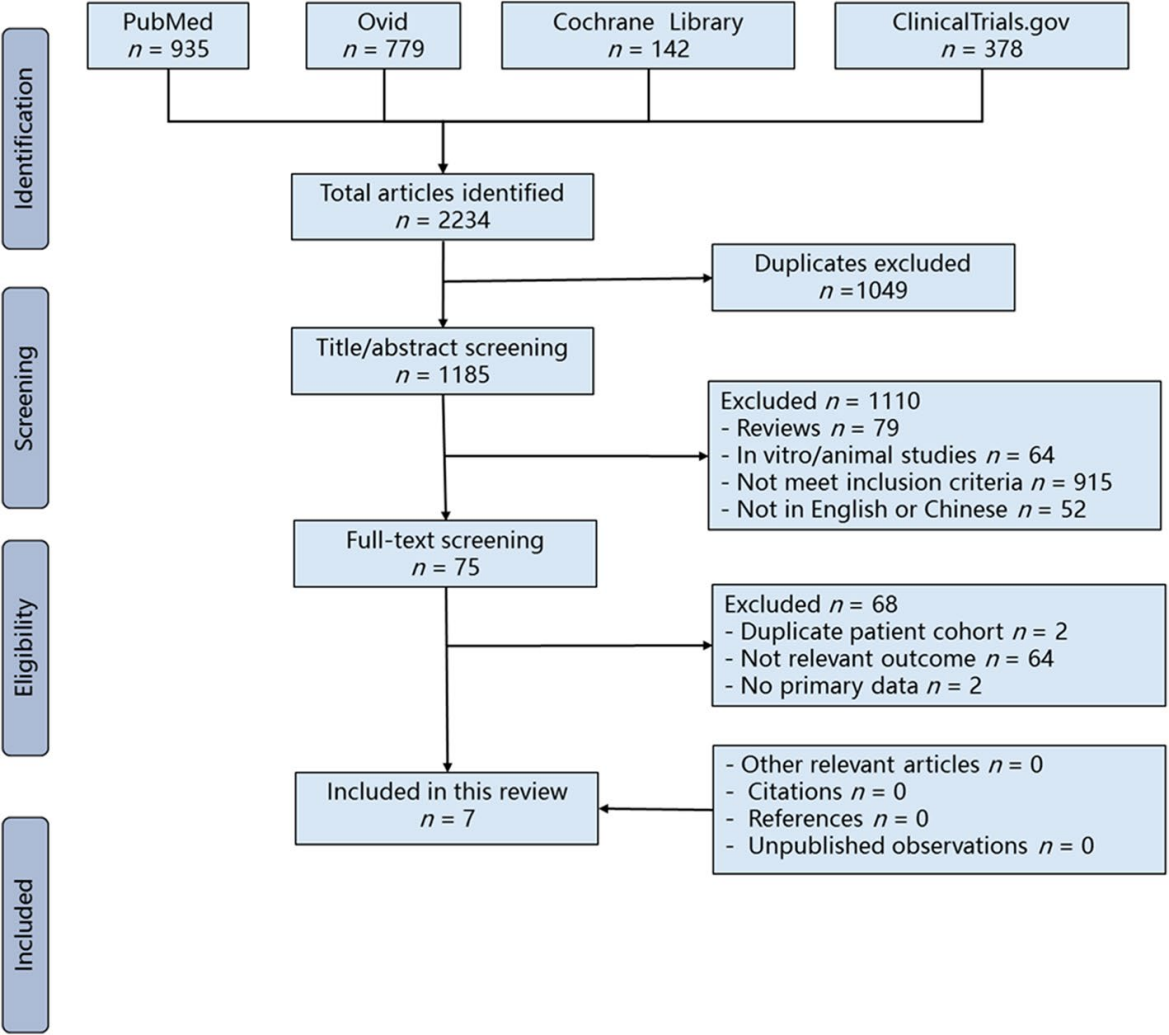
PRISMA flowchart of included studies

After the initial search, two principal investigators (QW Wu and S Chen) independently screened the titles and abstracts according to predefined inclusion and exclusion criteria. The eligibility criteria were as follows: ⑴ studies assessing the outcomes of CAL in breast cancer patients after radiotherapy, ⑵ studies expressly stating the methodology of CAL and recurrence outcomes, ⑶ studies with complete follow-up (at least 3 months), ⑷ studies involving humans regardless of whether they included a control group due to the limited number of clinical studies in the area, and ⑸ articles written in English or Chinese with full text. However, studies that only contained a history of lipofilling neither enriched with ADSCs nor SVF or only described the concept or protocol were excluded. Potentially relevant articles and those with insufficient information in the title and abstract were retrieved for full-text review. The two investigators then independently screened the full-text articles. Disagreements were resolved by consensus. The PRISMA flow diagram (Fig. 1) shows the entire review process, from the original search to the final selection of studies.

### Data extraction

The data items extracted from each included study are listed in Tables 2 and 3. Oncological safety was assessed through the number of cancer recurrences found in patients from individual studies. The primary outcome measures were the locoregional recurrence rates, which were considered the most relevant to the oncological safety of local treatment with CAL. The data collected were reported individually or combined as ranges for a particular variable without any assumptions. Due to the heterogeneity of these selected studies, including protocol design, patient characteristics, radiotherapy information, and outcomes measured, a formal meta-analysis of the data was not possible.

**Table 2:**
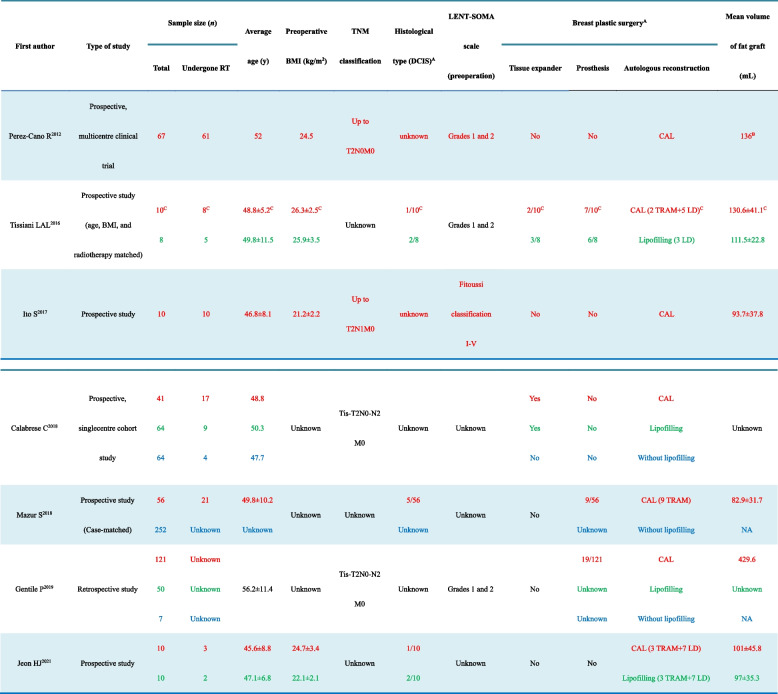
Characteristics of the studies included in the systematic review

*RT* Radiotherapy, *BMI* Body mass index, *DCIS* Ductal carcinoma in situ, *LENT-SOMA* the late effects normal tissues-subjective objective management analysis scoring system, *CAL* Cell-assisted lipotransfer, *TRAM* Transverse rectus abdominis myocutaneous flap, *LD* Latissimus dorsi flap, *NA* Not available. *A* below the oblique bar is the total sample size unless otherwise stated, *B* mean graft volume of the total two treatments, and *C* the nontumor case was deducted. The text in red represents the CAL group, the text in green represents the lipofilling group, and the text in blue represents the control group untreated with lipofilling

**Table 3:**
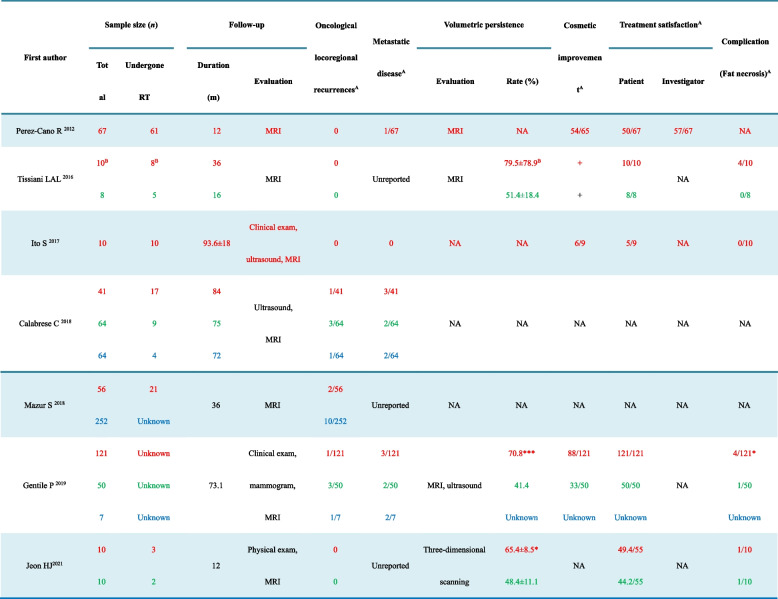
Outcomes of the participants included in the systematic review

*RT* Radiotherapy, *MRI* Magnetic resonance imaging, *NA* Not available

*A* below the oblique bar is the total sample size unless otherwise stated and *B* the nontumor case was deducted. The text in red represents the cell-assisted lipotransfer group, the text in green represents the lipofilling group, and the text in blue represents the control group untreated with lipofilling. *P < 0.05 and *** *P* < 0.001 versus the lipofilling group

## Results

### Literature search

The literature search initially yielded 2234 studies, including 378 clinical trials (Fig. 1). After the removal of duplicates, 1185 unique records were screened based on the titles and abstracts. Of these, 75 articles were screened based on the full texts. Ultimately, a total of seven studies, including two registered clinical trials (NCT00616135 and NCT01771913), published from 2012 to 2021, met all the inclusion and exclusion criteria of this systematic review (Fig. 1) [27–33].

### Study characteristics

Table 2 shows the general characteristics of the included studies. There were six prospective studies [27–31, 33], four of which had one or two control groups; and one retrospective study that had two control groups [32]. In Tissiani’s study, to control the risk of bias, stratified block randomization was performed to evenly distribute patients with radiotherapy [28]. Moreover, they started with patient selection in the CAL group, followed by the control group; the two groups were matched by age, BMI, and radiotherapy [28]. A stratified blocked randomization was also performed to evenly distribute the irradiated patients to the three groups in Gentile’s study [32]. Otherwise, no random allocation method was used in the other five studies to assign study subjects [27, 29–31, 33]. The risks of other bias in the included studies are as follows: First, most studies focused more on the outcomes of CAL without providing sufficient radiotherapy information, such as the method or dose used. Second, most of the studies were based on subjective questionnaires to investigate their satisfaction with the treatment outcomes in terms of three or five possible responses, but with no option to report any potential negative outcomes [27–29, 32, 33], demonstrating a possible element of bias. Third, the occurrence of postoperative complications in the relevant studies might be associated with the personal experience and skills of the surgeons; thus, the results could be biased in the relevant studies.

All studies enrolled female patients who underwent CAL in the context of breast reconstruction after radiotherapy. But only Ito’s study solely enrolled irradiated patients underwent breast reconstruction with CAL, with a sample size of 10 [29]. The studies of Perez-Cano solely enrolled patients who underwent breast reconstruction with CAL; the sample size was 67, and the ratio of irradiated patients was 91.0% (61/67) [27]. Tissiani’s and Jeon’s studies both set up the CAL group and the lipofilling control group (fat graft without ADSC enrichment, also known as the conventional lipofilling group) [28, 33]. Mazur’s study had the CAL group and the control group untreated with lipofilling [31]. The remaining two studies had three groups: the CAL group, the lipofilling control group, and the control group untreated with lipofilling [30, 32].

### Participants

As shown in Table 2, the mean age of the participants was under 60 years. The mean preoperative body mass index was less than 30 kg/m^2^, except that was not mentioned in the other three studies [30–32]. The TNM classification of the tumor was up to T2N2M0 [27, 29, 30, 32]. Only three studies reported the histological type of tumor; the ratio of ductal carcinoma in situ was 10% (1/10), 8.9% (5/56), and 10% (1/10) in the CAL group of Tissiani’s, Mazur’s, and Jeon’s studies [28, 31, 33], while it was 25% (2/8) and 20% (2/10) in the lipofilling control group in Tissiani’s and Jeon’s studies, respectively [28, 33].

Perez-Cana et al. and Ito et al. stated the patients’ radiation history, which had a mean cumulative dose of 60 and 50.9 Gy, respectively [27, 29]. However, the method of radiotherapy technique used was not reported in all seven studies. The Late Effects Normal Tissues—Subjective Objective Management Analysis (LENT-SOMA) scoring system [34] was used to assess the physical symptoms and function damage from radiotherapy in Perez-Cana’s, Tissiani’s, and Gentile’s studies, which only enrolled patients with grades 1 and 2 [27, 28, 32], while the Fitoussi classification system was used in Ito’s study [29].

### Intervention (technical factors)

All participants in the included studies had undergone mastectomy or breast-conserving surgery. One study reported that a tissue expander temporary prosthesis was used before lipofilling when performing nipple-sparing mastectomy [30]. Tissiani et al., Mazur et al., and Gentile et al. reported that the ratio of prosthesis-based reconstruction in patients underwent CAL was 70% (7/10), 16.1% (9/56), and 15.7% (19/121) [28, 31, 32]. In addition, Tissiani et al., Mazur et al., and Jeon et al. enrolled participants who had undergone breast reconstruction with autologous flaps, either transverse rectus abdominis myocutaneous, or latissimus dorsi flaps [28, 31, 33]; the ratio of autologous flap-based reconstruction in the CAL group was 70% (7/10), 16.1% (9/56), and 100% (10/10), while the ratio was 37.5% (3/8) and 100% (10/10) in the lipofilling control group of Tissiani’s and Jeon’s studies [28, 33].

According to Coleman’s method, lipoaspirate was harvested from the abdominal region of patients [35]. Four studies applied the automated Celution® system (Cytori Therapeutics, San Diego, CA, USA) with a proteolytic enzyme to obtain ADSC-enriched grafts [27, 29, 30, 32], while the other three studies used collagenase [28, 31, 33]. The volume of harvested adipose varied across the studies. The mean volume of ADSC-enriched grafts was reported in five studies, which ranged from 82.9 to 136 mL [27–29, 31, 33]; while an average of 429.6 mL ADSC-enriched grafts was used in Gentile’s study [32], which was not stated in Calabrese’s study [30]. Tissiani et al. reported that the ratio of the adipose tissue needed for ADSC enrichment versus that needed for final injection was 2:1 [28], while that in the remaining six studies was 1:1. On the other hand, Ito et al., Mazur et al., and Gentile et al. reported that the cell number ranged from one hundred thousand to a million cells per milliliter ADSC-enriched graft [29, 31, 32]. Tissiani et al., Mazur et al., and Gentile et al. detected the immunophenotype and stem cell characterization of ADSCs [28, 31, 32].

### Volumetric persistence (fat graft retention)

The breast volume was monitored by ultrasound, magnetic resonance imaging (MRI), or three-dimensional surface imaging [28, 32, 33]. Tissiani et al. reported that the volumetric persistence in the CAL group was higher (79.5% ± 78.9%) than that in the lipofilling group (51.4% ± 18.4%); however, the difference was statistically significant (*P* = 0.31) [28]. In Gentile’s and Jeon’s studies, volumetric persistence was higher in the CAL group than in the lipofilling group (*P* < 0.05) [32, 33]. Briefly, from the limited evidence, breast reconstruction with CAL had higher volumetric persistence than conventional lipofilling.

### Aesthetic improvement, treatment satisfaction, and complications

Aesthetic improvement was assessed by clinical evaluation, including MRI, ultrasound, and surgeon peer analysis. As shown in Table 3, after more than 12 months of follow-up, most participants presented aesthetic improvements [27–29, 32]. Based on either the LENT-SOMA scale assessment [27] or satisfaction assessment questionnaire [28, 29, 32, 33], most available patients [27–29, 32, 33] and investigators [27] were satisfied with the treatment results. There were no serious adverse events associated with the CAL procedure, such as disease transmission or septicaemia resulting from bacterial contamination [36–38]. Fat necrosis was reported to be the most common complication in the three studies of Tissiani et al., Gentile et al., and Jeon et al.; neither the incidence rates between the CAL group and the lipofilling group were significantly different (*P* > 0.05) [28, 32, 33]. Therefore, reconstruction with CAL presented aesthetic improvement and had favorable satisfaction but did not have adverse complications.

### Oncological safety and efficacy in irradiated patients

All seven studies enrolled patients who underwent breast reconstruction with CAL after radiotherapy. The follow-up duration ranged from 12 to 93.6 months. As shown in Table 3, neither loco-regional recurrence nor metastatic disease was observed in the three studies of Tissiani et al., Ito et al., and Jeon et al. [28, 29, 33]. Mazur et al. reported that the oncological recurrence rate of the CAL group was 3.6% (2/56), which did not differ significantly from that of the control group (10/252, 4.0%; *P* > 0.05) [31]. Thus, CAL did not increase recurrence risk following radiotherapy during the 3-year observation [31]. In the longer follow-up of C. Calabrese’s study [30], the loco-regional recurrence rate was 2.4% (1/41), 4.7% (3/64), and 1.6% (1/64) in the CAL group, the lipofilling group, and the control group untreated with lipofilling, respectively; and the ratio of systematic recurrence was 7.3% (3/41), 3.1% (2/64), and 3.1% (2/64), respectively. Thus, breast reconstruction with CAL did not increase oncological recurrence after nipple-sparing mastectomy [30]. Similarly, in Gentile’s study, the ratio of loco-regional recurrence and systematic recurrence in the three groups were 0.8% (1/121), 6% (3/50), 14.3% (1/7), and 2.5% (3/121), 4.0% (2/50), and 28.6% (2/7), respectively; CAL was also found to be oncologically safe in breast cancer patients [32]. Perez-Cano et al. reported that there was no local cancer recurrence, but one of the 67 patients had pelvic bone metastasis that was considered unrelated to CAL treatment during the 12-month follow-up [27]. Therefore, all of the above studies supported that CAL did not increase recurrence risk in breast cancer patients; ADSC-enriched fat grafts were oncologically safe in a long-term follow-up.

Then, we sorted out the patients’ demographics that were presented in the studies of Tissiani et al., Ito et al., and Jeon et al. in detail [28, 29, 33]. Since irradiated patients were not found to have any locoregional recurrence or metastatic disease during at least 12 months of follow-up, breast reconstruction with CAL was considered to be safe for irradiated patients [28, 29, 33]. Furthermore, based on whether had undergone radiotherapy, patients reconstructed with CAL were divided into two groups: irradiated patients reconstructed with CAL and nonirradiated patients reconstructed with CAL (Table 4). In Jeon’s study [33], the mean volumes of fat grafts in these two groups were 146.7 ± 46.2 mL and 81.4 ± 28.5 mL, respectively (*P* < 0.05), and the rates of volumetric persistence were 55.2 ± 11.0% and 69.8 ± 4.2%, respectively (*P* < 0.05). These results indicated that compared to the nonirradiated patients, irradiated patients reconstructed with CAL might need more adipose transplantation but had lower fat graft retention.

**Table 4:** Patient demographics extracted from the three studies (irradiated patients reconstructed with CAL vs. nonirradiated patients reconstructed with CAL)

First author	Age (years)	Preoperative BMI (kg/m^2^)	Tumor type	Type of reconstruction	Volume of fat graft (mL)	Follow-up (m)	Volumetric persistence (%)
**Irradiated patients reconstructed with CAL**							
**Tissiani LAL **^**2016**^							
	55	24.2	LCI	LD + IMPL	45	49	-27.33
	47	25.4	Mucinous	LD + IMPL	92	45	68.17
	48	27.2	DCI	LD + IMPL	137	38	87.20
	41	27.5	DCIS	TRAM	147	37	276.51
	54	24.0	DCI	LD + IMPL	141	34	69.07
	44	23.5	DCI	LD + IMPL	117	33	31.97
	56	23.9	DCI	TRAM	111	33	99.79
	43	30.9	LCI	Seq-explantation	159	20	31.38
mean ± SD	48.5 ± 5.8	25.8 ± 2.6	-	-	118.6 ± 36.8	36.1 ± 8.7	79.6 ± 89.0
**Ito S **^**2017**^							
	56	21.1	NA	No	120	NA	NA
	56	25.3	NA	No	80	NA	NA
	46	18.4	NA	No	154	NA	NA
	34	19.8	NA	No	84	NA	NA
	53	22.3	NA	No	83	NA	NA
	52	24.4	NA	No	98	NA	NA
	41	19.7	NA	No	50	NA	NA
	45	19.7	NA	No	46	NA	NA
	50	21.6	NA	No	152	NA	NA
	35	20.1	NA	No	70	NA	NA
mean ± SD	46.8 ± 8.1	21.2 ± 2.2	-	-	93.7 ± 37.8	-	-
**Jeon HJ **^**2021**^							
	35	20	DCI	LD	120	12	64.1
	46	22.9	DCI	TRAM	200	12	58.5
	36	30.1	DCI	LD	120	12	42.9
mean ± SD	39.0 ± 6.1	24.3 ± 5.2	-	-	146.7 ± 46.2*	12	55.2 ± 11.0*
**Nonirradiated patients reconstructed with CAL**							
**Tissiani LAL **^**2016**^							
	49	28	DCI	EXP + IMPL	177	38	63.4
	51	28.7	DCI	EXP + IMPL	180	37	95
**Jeon HJ **^**2021**^							
	54	26.8	DCI	LD	100	12	68.2
	44	22.7	DCI	LD	50	12	71.1
	49	29.6	DCI	TRAM	60	12	66.4
	64	22.6	LCI	LD	40	12	69.7
	48	26.3	DCIS	LD	120	12	74.6
	42	21.3	DCI	TRAM	100	12	62.8
	38	24.2	DCI	LD	100	12	75.7
mean ± SD	48.4 ± 8.0	24.8 ± 2.7	-	-	**81.4 ± 28.5**	12	**69.8 ± 4.2**

*CAL* Cell-assisted lipotransfer, *BMI* Body mass index, *LCI* Lobular carcinoma invasive, *DCI* Ductal carcinoma invasive, *DCIS* Ductal carcinoma in situ, *LD* Latissimus dorsi flap, *IMPL* Implant, *TRAM* transverse rectus abdominis myocutaneous flap, *EXP* Expander, *y* Year, *m* Months, *NA* Not available. * *P* < 0.05

To further confirm the safety and efficacy of CAL in irradiated patients, patients in the studies of Tissiani et al., Ito et al., and Jeon et al. were also sorted into two groups: irradiated patients reconstructed with CAL and irradiated patients reconstructed with lipofilling [28, 29, 33]. As shown in Table 5, the irradiated patients reconstructed with CAL in Tissiani’s study had an average longer follow-up than the irradiated patients reconstructed with lipofilling (36.1 ± 8.7 months vs. 13.8 ± 4.8 months, *P* < 0.01) [28]. After the follow-up, the mean volumetric persistence in the two groups was 79.6 ± 89.0% and 48.9 ± 19.4%, respectively; although the CAL group had higher volumetric persistence, the difference between the two groups was not statistically significant (*P* > 0.05). Moreover, the incidence of fat necrosis, the main complication in Tissiani’s study [28], was 50% (4/8) in the irradiated patients reconstructed with CAL, but none was observed in the irradiated patients reconstructed with lipofilling (0/5); the difference was not statistically significant (*P* > 0.05). Therefore, although ADSC-enriched fat grafts were oncologically safe in patients after breast radiotherapy; breast reconstruction with CAL did not have a higher rate of graft retention than conventional lipofilling.

**Table 5:** Demographics of patients who received radiotherapy extracted from the three studies (irradiated patients reconstructed with CAL vs. irradiated patients reconstructed with lipofilling)

First author	Age (y)	Preoperative BMI (kg/m^2^)	Tumour type	Type of reconstruction	Volume of fat graft (mL)	Follow-up (m) **	Volumetric persistence (%)
**Irradiated patients reconstructed with CAL**							
**Tissiani LAL **^**2016**^							
	55	24.2	LCI	LD + IMPL	45	49	-27.33
	47	25.4	Mucinous	LD + IMPL	92	45	68.17
	48	27.2	DCI	LD + IMPL	137	38	87.20
	41	27.5	DCIS	TRAM	147	37	276.51
	54	24.0	DCI	LD + IMPL	141	34	69.07
	44	23.5	DCI	LD + IMPL	117	33	31.97
	56	23.9	DCI	TRAM	111	33	99.79
	43	30.9	LCI	Seq-explantation	159	20	31.38
mean ± SD	48.5 ± 5.8	25.8 ± 2.6	-	-	118.6 ± 36.8	36.1 ± 8.7**	79.6 ± 89.0
**Ito S **^**2017**^							
	56	21.1	NA	No	120	NA	NA
	56	25.3	NA	No	80	NA	NA
	46	18.4	NA	No	154	NA	NA
	34	19.8	NA	No	84	NA	NA
	53	22.3	NA	No	83	NA	NA
	52	24.4	NA	No	98	NA	NA
	41	19.7	NA	No	50	NA	NA
	45	19.7	NA	No	46	NA	NA
	50	21.6	NA	No	152	NA	NA
	35	20.1	NA	No	70	NA	NA
mean ± SD	46.8 ± 8.1	21.2 ± 2.2	-	-	93.7 ± 37.8	-	-
**Jeon HJ **^**2021**^							
	35	20	DCI	LD	120	12	64.1
	46	22.9	DCI	TRAM	200	12	58.5
	36	30.1	DCI	LD	120	12	42.9
mean ± SD	39.0 ± 6.1	24.3 ± 5.2	-	-	146.7 ± 46.2	12	55.2 ± 11.0
**Irradiated patients reconstructed with lipofilling**							
**Tissiani LAL **^**2016**^							
	56	32.4	DCI	Seq-explantation	147	21	41.63
	69	25.9	DCIS	LD + IMPL	111	15	68.6
	38	24.1	DCI	LD + IMPL	108	13	70.23
	36	24.1	DCI	LD + IMPL	115	12	27.65
	49	24.9	DCIS	Seq-explantation	102	8	36.2
mean ± SD	49.6 ± 13.6	26.3 ± 3.5	-	-	116.6 ± 17.6	**13.8 ± 4.8**	48.9 ± 19.4
**Jeon HJ **^**2021**^							
	44	22.8	DCI	LD	60	12	36.2
	55	23.6	DCIS	LD	160	12	33.5

*CAL* Cell-assisted lipotransfer, *BMI* Body mass index, *LCI* Lobular carcinoma invasive, *DCI* Ductal carcinoma invasive, *DCIS* Ductal carcinoma in situ, *LD* Latissimus dorsi flap, *IMPL* Implant, *TRAM* Transverse rectus abdominis myocutaneous flap, *y* Year, *m* Months, *NA* Not available. **P < 0.01

## Discussion

In the present systematic review, we focused on the studies that evaluated the outcomes of breast cancer patients reconstructed with CAL after radiotherapy, and seven studies were eligible [27–33]. Based on the limited outcome evidence, the results of this study showed that CAL had oncological safety and did not increase recurrence risk in patients after breast radiotherapy. In irradiated patients, CAL does not have higher graft retention than conventional lipofilling; but more adipose tissue is needed to transplant. To the best of our knowledge, this is the first systematic review to evaluate the safety and efficacy of CAL in irradiated breasts.

Radiation is a component of breast cancer treatment and is especially recommended in postmastectomy patients with positive axillary lymph nodes or with negative nodes but tumors greater than 5 cm or positive pathologic margins [2]. It is also a mainstay of breast conservation surgery and offers a clear benefit in younger patients [2, 39]. With overall increasing survival rates and aesthetic pursuit [25, 40], the demand for postoperative breast reconstruction is rising. In particular, the psychological benefits have been broadly recognized, and breast reconstruction has become a component of neoplastic treatment [41]. Reconstructive techniques include implant-based reconstruction, reconstruction using autologous tissue, or both. Based on long-term experiences, lipofilling has been recognized as a safe and effective adjunct to breast reconstructive techniques and has also been found to be a popular stand-alone approach for breast reconstruction [5–7]. Adipose is a safe, neutral biological material that is easily accessible and able to be used to modify the body contour. Lipofilling can improve the results of implant-based reconstruction, especially if the expander or the implant is planned to be exchanged. It has a protective effect on recurrent infection, contracture, and fibrosis after radiotherapy [42, 43]. Two kinds of surgical procedures for lipofilling were developed according to the stuffing: the simple purification of lipoaspirate (conventional lipofilling) and lipoaspirate with ADSC enrichment (CAL). The former procedure was first established by Coleman et al. [35] and was performed by liposuction from a fatty area of the body (usually the abdomen or thighs). The specimen is purified by soft centrifugation to discard the oil and blood cells and then reinjected into the area to be reshaped but does not modify the concentration of ADSCs. In contrast, the enrichment technique needs to divide the lipoaspirate into two parts. The volumetric ratio of adipose for these two parts is usually 1:1. The first part is reserved for the final injection. The second part is processed by enzymatic digestion or mechanical separation to destroy the adult adipocytes; thus, ADSCs are concentrated. Then, the two parts of the specimen are mixed and reinjected into the area to be reshaped [12].

Supporters of the enrichment technique argue that ADSC enrichment favors the regenerative process of the recipient tissues and decreases the reabsorption risk of fat grafts [44] and demonstrate that ADSCs could reverse radiotherapy-induced tissue damage and chronic pain [45]. The possible mechanisms include their effects on the extracellular matrix, angiogenesis, and the inflammatory response [45]. Thus, these stem cells have potential applications in regenerative medicine, especially in irradiated tissue. However, many concerns have been raised about ADSCs increasing oncological risk in cancer patients [19–21]. The U.S. Food and Drug Administration warns that some patients may be vulnerable to stem cell treatments that are illegal and potentially harmful [46]. Thus, although CAL was first proposed more than ten years ago, it has not been widely used in breast cancer patients until recently. As the safety of CAL in breast cancer reconstruction has gradually been confirmed, radiotherapy in breast reconstruction with CAL appears to be a diminishing relative contraindication [47]. In the present study, our results demonstrated that CAL did not increase recurrence risk; it was oncologically safe in breast cancer patients after radiotherapy.

Notably, the results of Tissiani’s study showed that, although in irradiated patients, reconstruction with CAL had higher volumetric persistence than conventional lipofilling, the difference was not statistically significant (*P* > 0.05) [28]. Since the irradiated patients reconstructed with CAL had longer follow-ups (Table 5, *P* < 0.05), whether the efficacy of CAL diminished over time still needs to be further explored. On the other hand, the ADSC enrichment rate in the study [28] was twofold that in other studies [27, 29–33]. The higher supplementation rate of enrichment did not significantly improve the volumetric persistence of fat grafts, but more extra adipose tissue was needed in the surgical procedure [28]. This is an important practical consideration for irradiated patients with low BMI, as the extra adipose tissue required for ADSC enrichment may not be counterbalanced by increased volumetric persistence [48]. Furthermore, Jeon et al. demonstrated that when reconstructing with CAL, irradiated patients needed more adipose tissue but had lower graft retention than nonirradiated patients [33]. However, the results of the basic study from Luan et al. showed that CAL improved graft retention in irradiated recipient sites and rescued radiation-induced skin changes in immunocompromised mice [13]. Thus, more high-level clinical trials and basic researches were still needed to clarify the divergence.

Limitations of this study include the small sample of participants and the high levels of bias risk found within the studies. A comprehensive search strategy was used, but relevant studies may have been missed or have yet to be formally published. Many studies claimed that ADSCs were used for adipose transplantation; however, ADSCs were not enriched in the grafts [49, 50], and the technique was not the so-called CAL. Finally, only seven studies met all of the criteria in this systematic review. Another limitation of this systematic review is the short follow-up times that were insufficient to assess the long-term implications of using CAL technology in irradiated breasts. There was significant heterogeneity between the studies in terms of research design, patient characteristics, radiotherapy information, and outcome estimates; thus, it was impossible to conduct a rigorous meta-analysis. Although similar clinical trials are ongoing, the difficulty in recruiting research subjects always leads to the withdrawal of the study (such as registered Clinical Trial NCT01801878).

In conclusion, this systematic review concluded that CAL had oncological safety and did not increase recurrence risk after breast radiotherapy. Compared to conventional lipofilling, CAL improved the volumetric persistence of fat grafts in breast cancer patients; however, the efficacy of these two surgical procedures was comparable in irradiated patients. This suggests that the efficacy of CAL reconstruction might be limited in irradiated women seeking aesthetic breast augmentation, because it doubles the amount of adipose tissue required without consistently improving the outcome. As there is not yet a recognized way to predetermine the potential costs, both monetary and patient satisfaction, and aesthetic outcomes must be weighed against the cost of ADSCs enrichment to conventional lipofilling before making clinical decisions for irradiated patients. High-quality multicentre prospective studies, especially randomized controlled trials with adequate follow-up periods and standardized protocols, are therefore warranted to better inform decision-making in this setting.

## Data Availability

All data generated or analyzed during this study are included in this published article.

## Abbreviations

CAL: Cell-assisted lipotransfer
ADSC: Adipose-derived stem cells
SVF: Stromal vascular fraction
PRISMA: Preferred Reporting Items of Systematic Reviews and Meta-Analyses
LENT-SOMA: Late Effects Normal Tissues-Subjective Objective Management Analysis
MRI: Magnetic resonance imaging
