# Correction: Current perspectives on cell-assisted lipotransfer for breast cancer patients after radiotherapy

**DOI:** 10.1186/s12957-023-03134-2

**Published:** 2023-08-22

**Authors:** Qiuwan Wu, Shuai Chen, Wuyun Peng, Donghan Chen

**Affiliations:** 1 The First Affiliated Hospital of Xiamen University, School of Medicine, Xiamen University, 55 Zhenhai Road, Siming District, Xiamen, Fujian 361003 P. R. China; 2 The Third Clinical Medical College, Fujian Medical University, Xiamen, Fujian P. R. China

**Correction: World J Surg Onc 21, 133 (2023)**

**https://doi.org/10.1186/s12957-023-03010-z**

Following publication of the original article [1], the author reported that the year citations of reference 33 in the maintext body were incorrect. It should be 2021 instead of 2020. The following occurences should be corrected.

**Results section**, Literature search, 4th sentence “published from 2012 to 2020,” should be “published from 2012 to 2021,”

In tables 2 to 4, “Jeon HJ 2020” should be “Jeon HJ 2021.”

The original article has been updated.
